# Pan-Cancer Molecular Characterization of m^6^A Regulators and Immunogenomic Perspective on the Tumor Microenvironment

**DOI:** 10.3389/fonc.2020.618374

**Published:** 2021-01-28

**Authors:** Jie Zhu, Jiani Xiao, Min Wang, Daixing Hu

**Affiliations:** ^1^Department of Intensive Care Unit, The People’s Hospital of Tongliang District, Chongqing, China; ^2^Department of Pediatric Dentistry, West China Hospital of Stomatology, Sichuan University, Chengdu, China; ^3^Department of Respiratory and Geriatrics, Chongqing Public Health Medical Center, Chongqing, China; ^4^Department of Urology, First Affiliated Hospital of Chongqing Medical University, Chongqing, China

**Keywords:** N6-methyladenosine methylation, tumor microenvironment, tumor-infiltrating lymphocytes, The Cancer Genome Atlas (TCGA), pancancer analysis

## Abstract

**Purpose:**

N6-methyladenosine (m^6^A) methylation plays a critical role in diverse biological processes. However, knowledge regarding the constitution of m^6^A on tumor microenvironment (TME) and tumor-infiltrating lymphocytes (TILs) across cancer types is still lacking. We performed comprehensive immuno-genomic analyses to reveal molecular characterization of the m^6^A regulators and immune-related genes (IRGs) across TME and TIL heterogeneity.

**Methods:**

We comprehensively analyzed the properties of m^6^A regulators in genomic profiles from The Cancer Genome Atlas (TCGA) according to expression perturbations of crucial IRGs, CD274, CD8A, GZMA, and PRF1. The four IRGs were proved to be reliable biomarkers of TILs and TME *via* CIBERSORT and ESTIMATE analyses, and their co-expression relationship was certified by TIMER analysis. Based on their median values, the samples from the pan-cancer tissues (N = 11,057) were classified into eight TME types. The RNA expression levels of 13 m^6^A regulators were compared across TME subtypes. Single-sample Gene Set Enrichment Analysis (ssGSEA) was also used to classify TME clusters, expression variants of IRGs and m^6^A regulators were verified among TME clusters. Meanwhile, the correlation between m^6^A regulators and tumor mutational burden (TMB) were tested. Finally, the impacts of IRGs and TME clusters in clinical characteristics and outcomes were revealed.

**Results:**

CD274, CD8A, GZMA, and PRF1 showed similar TILs’ characteristics, of which the level of T cells CD8 and T cells CD4 memory activated are consistent with the expression levels of the four IRGs and higher immune infiltration. Besides, CD274, CD8A, GZMA, and PRF1 were positively correlated with the stromal score or immune score in almost all 33 tumor types. All of four IRGs showed impact between tumor pathological stages or clinical outcomes. Among TME type I to type IV, m^6^A regulators’ expression drift changed from high-level to low-level in ESCA, BLCA, HNSC, CESC, BRCA, and GBM. However among TME type V to type VIII, m^6^A regulators drew a shift from low-level to high-level expression in CESC, BLCA, ESCA, KIRP, HNSC, BRCA, KIRC, COAD, LAML, GBM, and KICH. In ssGSEA analyses, IRGs’ expression levels were elevated with the immune infiltration degree and m^6^A regulators’ expression level varied among three TIL subgroups. With different TMB levels, expression differences of m^6^A regulators were observed in BLCA, BRCA, COAD, LGG, LUAD, LUSC, STAD, THCA, and UCEC.

**Conclusion:**

We identified four crucial IRGs affecting TILs, TME characteristics and clinical parameters. Expression variants of m^6^A regulators among the subgroups of TME types and ssGSEA clusters suggested that m^6^A regulators may be essential factors for phenotypic modifications of IRGs and thus affecting TME characteristics across multiple tumor types.

## Introduction

Immunotherapy, as a viable treatment for multiple cancers, has recently received extensive attention. T cell-based immunotherapy has been called as immune checkpoint inhibitors (ICIs), such as anti-cytotoxic T-lymphocyte-associated protein 4 (CTLA4), anti-programmed death protein-1 (PD-1), or anti-programmed death-ligand 1 (PD-L1) antibodies (1–3). Immune-related treatments targeting T cell exhaustion markers can improve cancer outcomes by enhancing antitumor immunity (1), which have shown significant clinical efficacy in immunogenic tumors such as melanoma, renal cell carcinoma, bladder cancer, non-small cell lung cancer, and hodgkin’s lymphoma (2, 4–6). However, not all patients respond well to ICIs therapy. The variable response is also associated with patients’ genomic characteristics such as tumor microenvironment (TME) and tumor mutation burden (TMB) (7–9). Accumulating researches have shown that tumor cells could change the TME to serve as contributors that ensure rapid cell proliferation (10). The dynamic alteration of molecular and cellular processes in TME relying on the interactions between tumor cells and immune cells (11), which highlights the role of tumor-infiltrating lymphocytes (TILs) in the context of protumorigenic inflammation and anticancer immuno-surveillance (12). Hence, researchers have attempted to analyze the detailed composition, density, and function of TILs in TME context, which turn out to be challenging.

Several studies have shown that the mRNA-seq value of some crucial immune-related genes (IRGs) could constitute appropriate models for assessing TME. Rooney et al. reported a quantitative measure of immune cytolytic activity (CYT) based on the expression levels of granzyme A (GZMA) and perforin 1 (PRF1), which was also a model to assess TME (13). A study used the median PD-L1 (assessed by CD274 expression) and CD8A expression levels as the cut-off values to define subgroups in TME, of which the response to ICIs treatment was proved to differ among subgroups (14). Another published research proposed to classify TME depending on PD-L1 status and presence or absence of TILs, which also indicated specific TILs with PD-L1 positive would benefit more from anti-PD-L1/PD-1 therapies (15).

In most eukaryotes, m^6^A methylation is the most abundant internal chemical modification around the 3′ untranslated region (3′ UTR) of mRNA (16). Protein complexes and related coding genes have been classified as methyltransferases (“writers”), binding proteins (“readers”), and demethylases (“erasers”). Based on current research, the writers mainly include WT1-associated protein (WTAP), methyltransferase like 3 (METTL3), methyltransferase like 14 (METTL14), RNA binding motif protein 15 (RBM15), zinc finger CCCH-type containing 13 (ZC3H13), and the readers include YTH domain-containing 1 (YTHDC1), YTH domain-containing 1 (YTHDC2), YTH N6-methyl-adenosine RNA binding protein 1 (YTHDF1), YTH N6-methyladenosine RNA binding protein 2 (YTHDF2), YTH N6-methyladenosine RNA binding protein 3 (YTHDF3) and heterogeneous nuclear ribonucleoprotein C (HNRNPC). The erasers contain fat mass- and obesity-associated protein (FTO) and *α*-ketoglutarate-dependent dioxygenase alkB homolog 5 (ALKBH5) (17–19). M^6^A methylation controls many mRNA features, such as structure formation, maturation, stability, splicing, export, translation, and decay (20). It also regulates cell fate, cell cycle arrest, cell differentiation, eventually leading to the occurrence of cancer (21, 22). Recently, it has been recognized as a crucial factor in T cell homeostasis (23). Selectively altered m^6^A regulator levels may be effective adjuvant therapy strategies in a variety of immunological diseases (24–26). But knowledge regarding the fluctuation of m^6^A regulators in TILs, TME, and immunotherapies has not been clearly elucidated. Research based on the heterogeneity of m^6^A regulators to identify distinct subtypes of sepsis (27), of which the GSEA and CIBERSORT analyses found different immunocompetent status (such as Th1 cells, T cells CD4 activated, NK cells activated and B cells activated) among subtypes and indicated the potential relation among m^6^A regulators and leukocyte infiltration. Studies also have shown that m^6^A regulators contribute to TME formation (28) and affect the abundance of TILs (29) as well as response to ICIs treatment (30).

In our study, we performed comprehensive immuno-genomic analyses to provide a thorough understanding of the m^6^A regulator alterations and IRGs expression perturbations across TME and TILs heterogeneity. We extracted the data of patients with 33 tumor types from The Cancer Genome Atlas (TCGA) database and systematically characterized them into subgroups depending on TME or TILs characteristics. We found specific and widespread genetic alteration patterns in m^6^A regulators and IRGs in this context. We also assessed the relationship between TMB and m^6^A regulators and explored the prognostic value of IRGs or TME clusters. Our analysis emphasizes the vital effect of m6A regulators on the crucial IRGs, which lays a foundation for further research to improve ICIs treatment strategies.

## Materials and Methods

### Data Availabilities

The original contributions presented in the study are publicly available in the TCGA database (https://portal.gdc.cancer.gov/). This data can be found in the UCSC Xena browser (https://xenabrowser.net);. Tumor gene expression data, TMB data, and corresponding clinical data, including survival time (overall survival, OS; disease-specific survival, DSS; progression-free interval, PFI), survival status, age, and tumor stages, as well as the somatic mutation (SNPs and small INDELs) data, were obtained across 33 tumor types. Data of TMB in this study were directly generated from the somatic mutation data. A total of 11,057 tumor samples in the TCGA cohort were included, and gene expression levels were presented as the log 2-transformed (FPKM+1) values.

### Cancer Types Investigated in this Study

Adrenocortical carcinoma (ACC), Bladder Urothelial Carcinoma (BLCA), Breast invasive carcinoma (BRCA), Cervical squamous cell carcinoma and endocervical adenocarcinoma (CESC), cholangiocarcinoma (CHOL), Colorectal adenocarcinoma (COAD), Diffuse large B-cell lymphoma (DLBC), Esophageal Carcinoma (ESCA), Glioblastoma multiforme (GBM), Head and Neck squamous cell carcinoma (HNSC), kidney chromophobe (KICH), Kidney renal clear cell carcinoma (KIRC), Kidney Renal Papillary Cell Carcinoma (KIRP), Acute myeloid leukemia (LAML), Brain Lower Grade Glioma (LGG), Liver hepatocellular carcinoma (LIHC), Lung adenocarcinoma (LUAD), Lung squamous cell carcinoma (LUSC), mesothelioma (MESO), Ovarian serous cystadenocarcinoma (OV), pancreatic adenocarcinoma (PAAD), Pheochromocytoma and Paraganglioma (PCPG), Prostate Adenocarcinoma (PRAD), rectum adenocarcinoma (READ), Sarcoma (SARC), Skin Cutaneous Melanoma (SKCM), Stomach adenocarcinoma (STAD), Testicular germ cell tumors (TGCT), Papillary Thyroid Carcinoma (THCA), Thymoma (THYM), Uterine Corpus Endometrial Carcinoma (UCEC), Uterine carcinosarcoma (UCS), Uveal Melanoma (UVM).

### Tumor Immune Estimation Resource

TIMER is a comprehensive resource (http://timer.cistrome.org/) that consists of six major analytic modules that allow users to explore the association of TILs abundance with gene expression, overall survival, somatic mutations, and DNA somatic copy number alterations (SCNAs), as well as analysis of differential gene expression (DiffExp) and gene–gene correlations (31, 32).

### CIBERSORT

The proportions of the 22 tumor-infiltrating immune cells from each sample were determined by using the “CIBERSORT” (R package) (33), and gene expression profiles were transformed into the proportion of 22 TILs, namely: B cells naive, B cells memory, Plasma cells, T cells CD8, T cells CD4 naive, T cells CD4 memory resting, T cells CD4 memory activated, T cells follicular helper, T cells regulatory (Tregs), T cells gamma delta, NK. cells resting, NK. cells activated, Monocytes, Macrophages M0, Macrophages M1, Macrophages M2, Dendritic cells resting, Dendritic cells activated, Mast cells resting, Mast cells activated, Eosinophils, and Neutrophils. The relative expression of 22 tumor-infiltrating immune cells in each sample was determined. Significant results (P < 0.05) were selected for subsequent analysis.

### Estimation of Stromal and Immune Cells in Malignant Tumor Tissues Using Expression Data Scores and Immune Subtype Analyses

ESTIMATE (Estimation of Stromal and Immune cells in Malignant Tumor tissues using Expression data) is a newly developed algorithm that takes advantage of the unique properties of the transcriptional profiles of cancer tissues to infer tumor cellularity as well as the different infiltrating normal cells (34). The algorithm imputes stromal and immune scores to predict the level of infiltrating stromal and immune cells based on specific gene expression signatures of stromal and immune cells. Stromal and immune scores were calculated by using the “estimate” package with default parameters.

The immune-related prognostic signature was generated by a previously conducted TILs study (9). The aforementioned previous study comprehensively described the immune landscape of >10,000 samples, comprising 33 different cancer types, and integrated 160 immune-related signatures containing 2,995 immune genes. Six immune subtypes were defined as Wound Healing (Immune C1), IFN-gamma Dominant (Immune C2), Inflammatory (Immune C3), Lymphocyte Depleted (Immune C4), Immunologically Quiet (Immune C5), TGF-beta Dominant (Immune C6), measuring immune infiltrates in TME.

### Gene Enrichment Analysis

Single-sample Gene Set Enrichment Analysis (ssGSEA) is used to analyze TME features. It is an extension of Gene Set Enrichment Analysis (GSEA), which calculates separate enrichment scores for each pairing of a sample and gene set (35). Pearson’s correlation coefficient was used to calculate the correlation of the ssGSEA scores across the gene sets. The ssGSEA scores for most immune cell populations obtained using the gene sets from Angelova et al. (36). Those with ssGSEA scores consistent with known immune cell markers were retained for the gene sets included in no less than two published studies. Finally, a total of 29 gene sets representing distinct immune cell populations were selected, and the ssGSEA scores of each were calculated across 11,057 samples in the pan-cancer cohort. The following 29 types of immune-related gene sets were obtained: aDCs, APC co-inhibition, APC co-stimulation, CCR, CD8+ T cells, Check-point, Cytolytic activity, DCs, HLA, iDCs, Inflammation-promoting, Macrophages, Mast cells, MHC class I, Neutrophils, NK cells, Parainflammation, pDCs, T cell co-inhibition, T cell co-stimulation, T helper cells, Tfh, Th1 cells, Th2 cells, TIL, Treg, Type I IFN Reponse, Type II IFN Reponse. In this manner, ssGSEA transforms a single sample’s gene expression profile to a gene set enrichment profile. The enrichment scores calculated by ssGSEA analysis were utilized to represent the relative abundance of TME infiltrating cells in each sample.

### Statistical Analyses

Statistical analyses and data plotting were performed using R program (3.6.2). Unless noted otherwise, Fisher’s exact and equal-variance t-tests were, respectively, used in group comparisons for categorical and continuous variables. Spearman’s correlation analysis test was used to analyzed the correlation relationship in different cancer types. A threshold of 0.05 was used to deem significance from p values of statistical tests.

## Results

### The Tumor-Infiltrating Lymphocytes’ Distribution Related to Immune-Related Genes

According to previous studies we mentioned above, CD274, CD8A, GZMA, and PRF1 were chosen as the crucial IRGs to represent TILs’ characteristics in TME. To evaluate whether the four IRGs could tell the TILs’ characteristics, we further focused on TILs’ distribution in patients with differential expression of CD274, CD8A, GZMA, and PRF1. By taking the median value as threshold and using CIBERSORT as TILs component analysis, T cells CD4 memory activated (in 26 tumor types) expressed the most extensive infiltration differences in pan-cancer tissues and followed by Macrophages M1 (in 24 tumor types), T cells CD8 (in 20 tumor types), Macrophages M0 (in 15 tumor types) between the high- and low-expression groups of CD274 (**Supplement Figure 1**). A comparable result could be observed in high- and low-expression groups of CD8A, T cells CD8 (in 31 tumor types), T cells CD4 memory activated (in 26 tumor types), Macrophages M1 (in 26 tumor types), Macrophages M0 (in 24 tumor types) were also identified as differential infiltrated TIL types (**Supplement Figure 2**). Further analyses suggested that the TILs’ features did not change much in high- and low-expression groups of GZMA and PRF1. T cells CD8 (31 tumor types *versus* 32 tumor types), T cells CD4 memory activated (26 tumor types *versus* 26 tumor types), Macrophages M1 (26 tumor types *versus* 23 tumor types), Macrophages M0 (23 tumor types *versus* 20 tumor types) turned to be the consistent TILs pattern of inter-group differences (**Supplement Figures 3** and **4**). Additionally, T cells CD8 and T cells CD4 memory activated were always co-expressed with gene expression level. The high-expression groups of CD274, CD8A, GZMA, and PRF1 always tended to show higher infiltration of T cells CD8 and T cells CD4 memory activated. On the contrary, Macrophages M0 always showed higher infiltration in low-expression groups of CD274, CD8A, GZMA, and PRF1. There were no consistencies observed in Macrophages M2, Macrophages M1, NK cells resting, and Mast cells activated in groups of various tumor types.

### Relationship Between Estimation of Stromal and Immune Cells in Malignant Tumor Tissues Using Expression Data Scores and Immune-Related Genes

The ESTIMATE immune score and stromal score were used to analyze the infiltration levels of immune cells and stromal cells in different tumors. The correlation between the four IRGs and immune or stromal score was analyzed by Spearman’s correlation analysis. The results suggested a surprising degree of consistency, CD274 (in 32 tumor types, except THYM, **Supplement Figure 5**), CD8A (in 32 tumor types, except UCS, **Supplement Figure 6**), GZMA (in 33 tumor types, **Supplement Figure 7**), and PRF1 (in 33 tumor types, **Supplement Figure 8**) were all positively correlated with the stromal score or immune score in almost all 33 tumor types, which gave us the basis for continued classification of samples according to the IRG characteristics.

### M^6^A Regulators Distribution Across Immune Subtype of Tumor Microenvironment and Relationship With Immune-Related Genes Across Cancer Types

**Figure 1:**
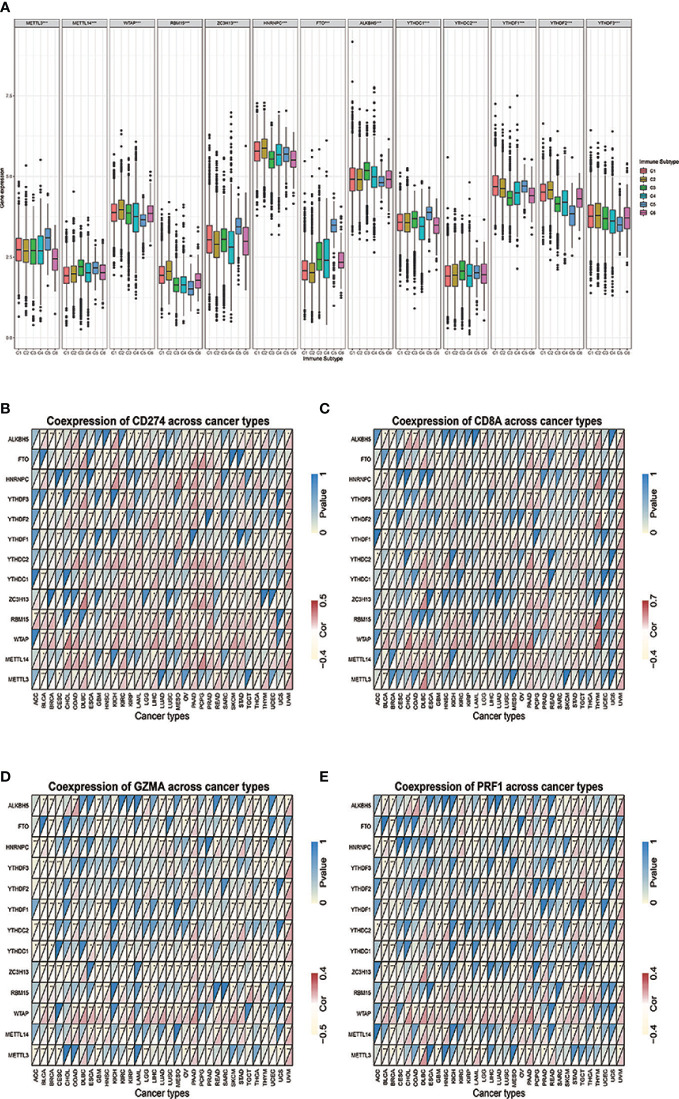
**(A)** The differential expression of m^6^A regulators was tested across the six immune subtypes (C1 to C6). **(B–E)** The correlations between IRGs and m^6^A regulators. * represents P < 0.05, ** represents P < 0.01, *** represents P < 0.001.

The differential expressions of m^6^A regulators were tested across the six immune subtypes (C1 to C6) reported by Thorsson, V. et al. METTL3, METTL14, WTAP, RBM15, ZC3H13, HNRNPC, FTO, ALKBH5, YTHDC1, YTHDC2, YTHDF1, YHDF2, and YTHDF3 were all significantly differentially expressed among six immune subtypes (p < 0.001) (**Figure 1**). Across 33 tumor types, CD274 and CD8A had broader positive correlations with m^6^A regulators (**Figures 1B, C****)**. By contrast, GZMA and PRF1 presented broader negative correlations (**Figures 1D, E****)**. By taking the median of the log 2-transformed (FPKM+1) values, we compared the expressions of m^6^A regulators between high- and low-expression groups of CD274 (**Supplement Figure 9**), CD8A (**Supplement Figure 10**), GZMA (**Supplement Figure 11**), and PRF1 (**Supplement Figure 12**) respectively. The results showed that m6A regulators’ levels had vast differences among these groups across various tumor types, of which KIRC, PAAD, and UVM are the top three tumor types showed the widest differences.

### M^6^A Regulator Distribution Across Immune-Related Gene Immune Types in Pan-Cancer Types

**Figure 2:**
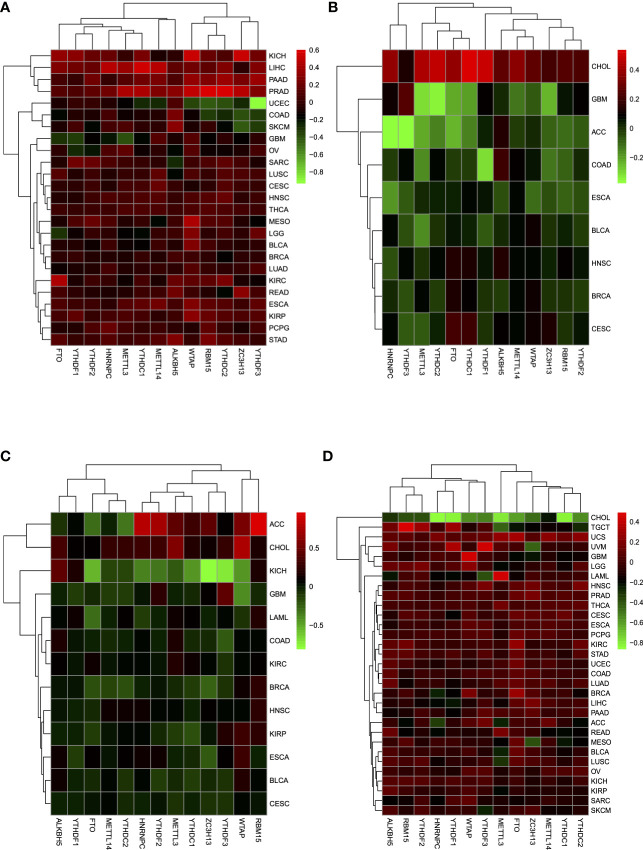
**(A)** Expression of m^6^A regulators between tumor tissues and normal tissues in type I and type II groups, which showed a higher expression tendency in tumor tissues. **(B)** In type III and type IV, m^6^A regulators tended to show a lower expression tendency in tumor tissues. **(C)** M^6^A regulators in tumor tissues showed a lower expression level between type V and type VI. **(D)** Between type VII and type VIII, m^6^A regulators showed a higher expression level in tumor tissues.

Through TIMER analysis, we observed positive correlations between CD274 and CD8A across 29 tumor types (more specifically, ACC, BLCA, BRCA, CESC, CHOL, COAD, DLBC, ESCA, HNSC, KIRC, KIRP, LGG, LIHC, LUAD, LUSC, MESO, OV, PAAD, PCPG, PRAD, READ, SARC, SKCM, STAD, TGCT, THCA, UCEC, UCS, UVM) (**Supplement Figure 13**). Moreover, GZMA and PRF1 also showed a tightly co-expressed correlation in 32 of the 33 tumor types (except for LAML) (**Supplement Figure 14**), which is consistent with the previous study (13). Based on the co-expression relationship of the four IRGs, it is suggested that by dividing four IRGs into two groups (CD274 and CD8A, GZMA, and PRF1, respectively), we may reveal a certain TME commonality and TILs similarity. After merging log 2-transformed values of the (FPKM+1) of CD274, CD8A, GZMA, and PRF1, we divided all of the TCGA samples into four groups as follows: type I, CD274 expression higher than the median and CD8A expression higher than the median; type II, CD274 expression higher than the median and CD8A expression lower than the median; type III, CD274 expression lower than the median and CD8A expression higher than the median; and type IV, CD274 expression lower than the median and CD8A expression lower than the median. Between type I and type II, tumor tissues showed higher m^6^A regulators expressions compared with normal tissues across multiple tumor types (**Figure 2A**). A similar analysis in type III and type IV revealed that m^6^A regulators turned to expressed in lower level (**Figure 2B**). More specifically, m^6^A regulators’ expression drift changed from high-level to low-level in ESCA, BLCA, HNSC, CESC, BRCA, and GBM.

In the same way, we divided all of the TCGA samples into four groups as follows: type V, GZMA expression higher than the median and PRF1 expression higher than the median; type VI, GZMA expression higher than the median and PRF1 expression lower than the median; type VII, GZMA expression lower than the median and PRF1 expression higher than the median; and type VIII, GZMA expression lower than the median and PRF1 expression lower than the median. Compared with type V and type VI, m^6^A regulators drew a shift from lower expression level to higher level in type VII and type VIII between tumor tissues and normal tissues in CESC, BLCA, ESCA, KIRP, HNSC, BRCA, KIRC, COAD, LAML, GBM, and KICH (**Figures 2C, D****)**. Only in CHOL, m^6^A regulators changed from higher to lower expression tendency. Overall, when we grouped the patients depending on the expression levels of IRGs, m^6^A regulators reflected dramatic contrast changes among groups. The considerable expression fluctuation among groups revealed that m^6^A regulators might be the crucial factors affecting IRGs expression and thus affecting TME immune infiltration.

### The Relevance of Immune-Related Genes and m^6^A Regulators With Tumor Microenvironment Features

**Figure 3:**
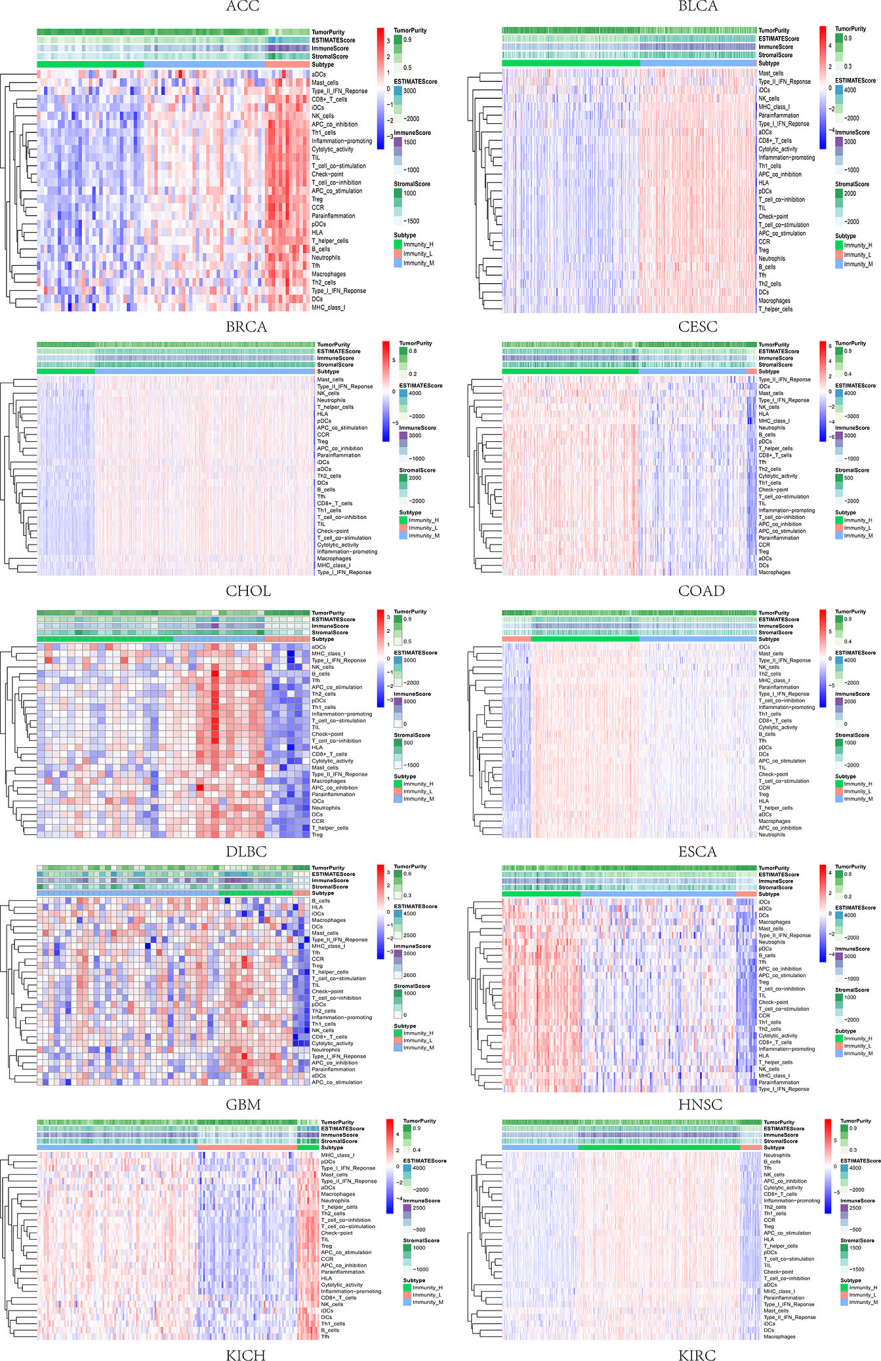
Three subgroups were defined as immunity-high, immunity-medium, and immunity-low by ssGSEA scores across 33 tumor types.

**Figure 4:**
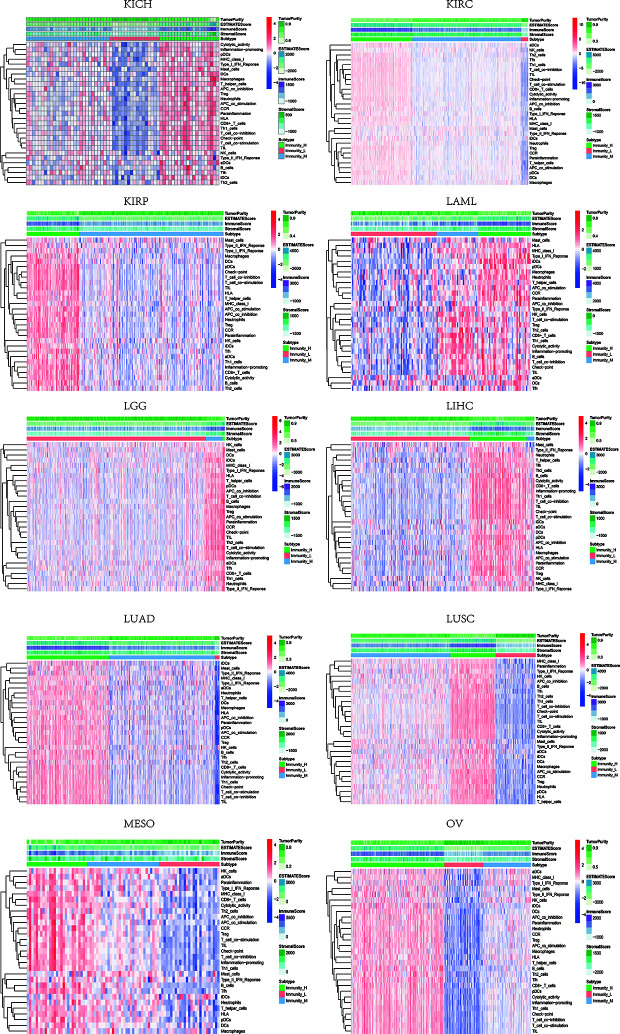
Three subgroups were defined as immunity-high, immunity-medium, and immunity-low by ssGSEA scores across 33 tumor types.

**Figure 5:**
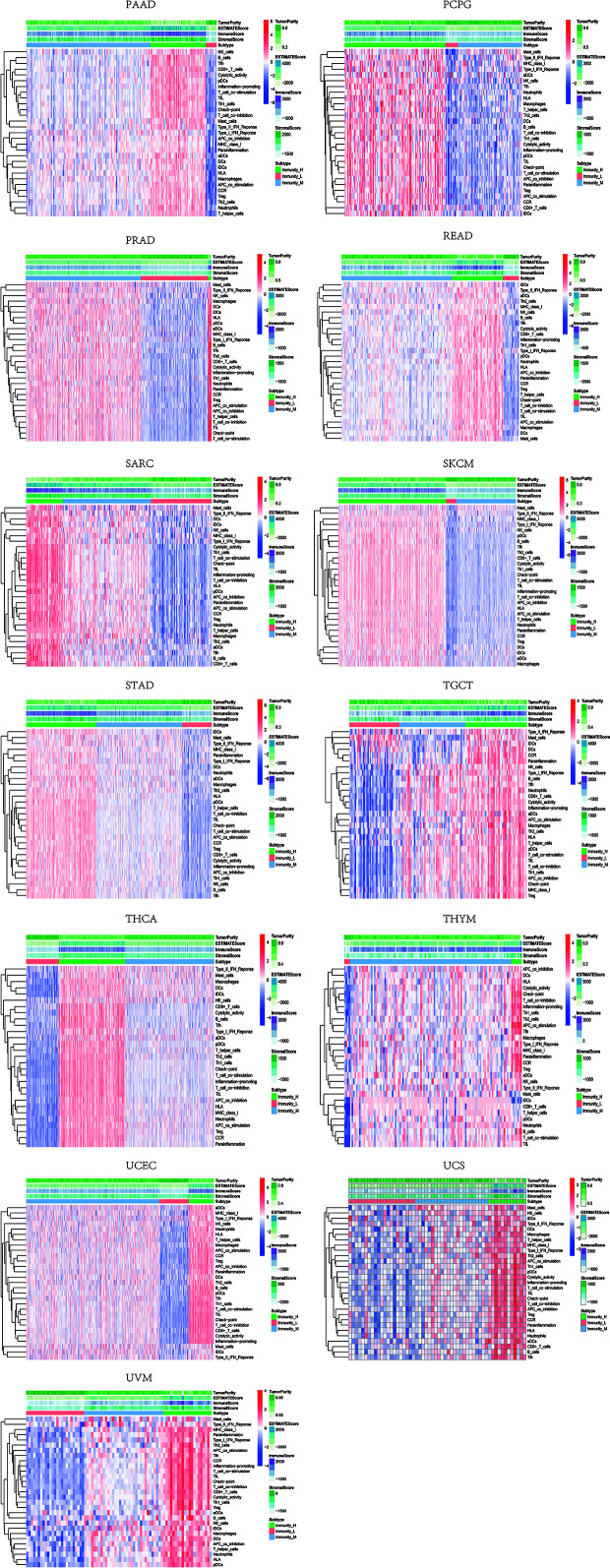
Three subgroups were defined as immunity-high, immunity-medium, and immunity-low by ssGSEA scores across 33 tumor types.

**Figure 6:**
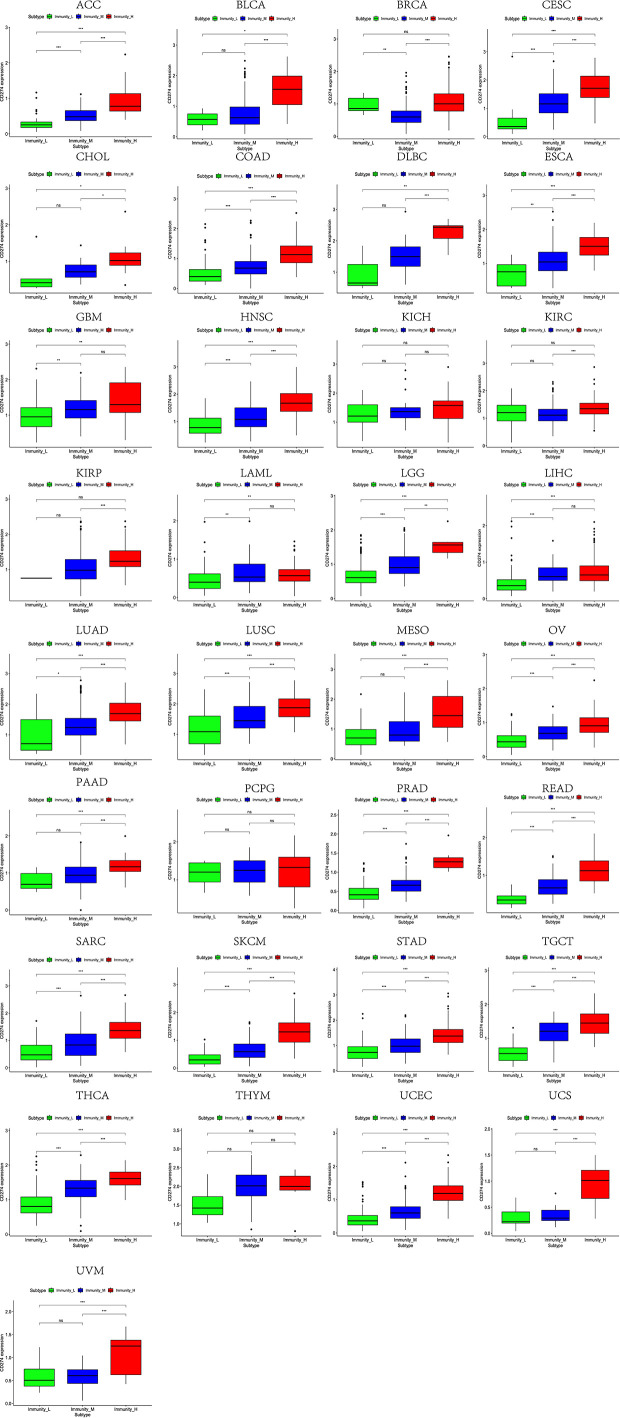
High expression level of CD274 was presented in high immune infiltration across 33 tumor types.

**Figure 7:**
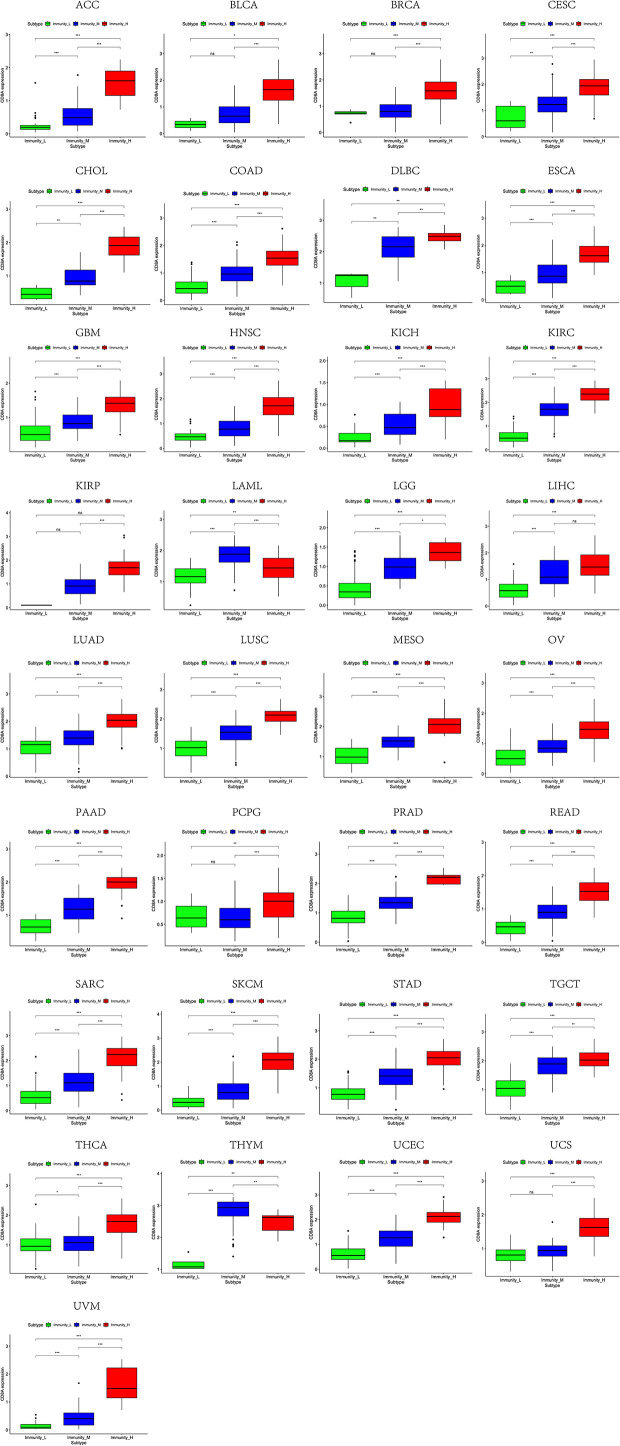
High expression level of CD8A was presented in high immune infiltration across 33 tumor types.

**Figure 8:**
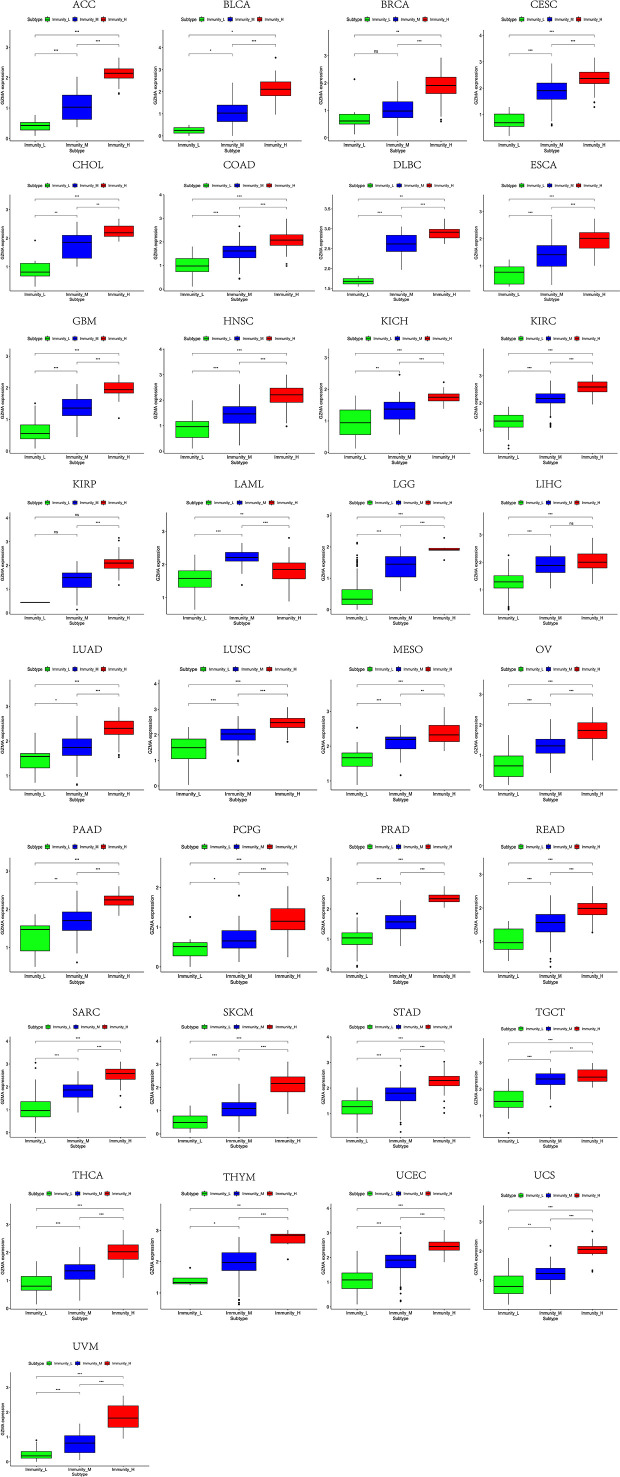
High expression level of GZMA was presented in high immune infiltration across 33 tumor types.

**Figure 9:**
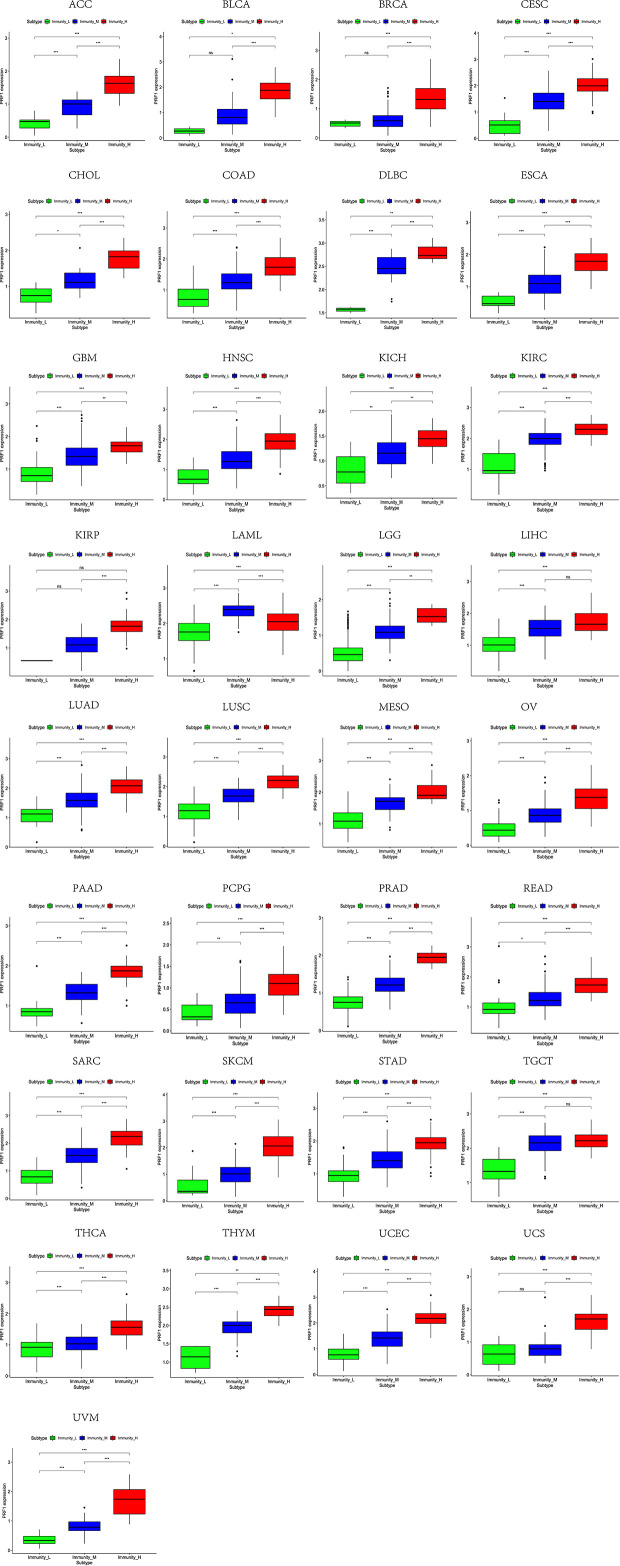
High expression level of PRF1 was presented in high immune infiltration across 33 tumor types.

**Figure 10:**
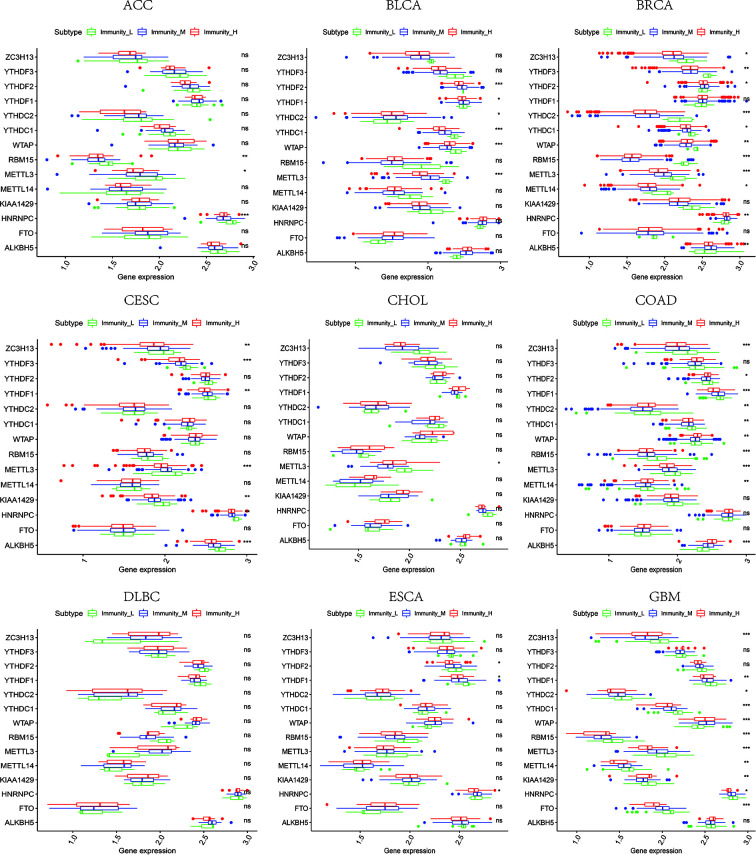
The expression diversity of m^6^A regulators among high, medium, and low immune infiltration levels. * represents P < 0.05, ** represents P < 0.01, *** represents P < 0.001, ns represents P ≥ 0.05.

**Figure 11:**
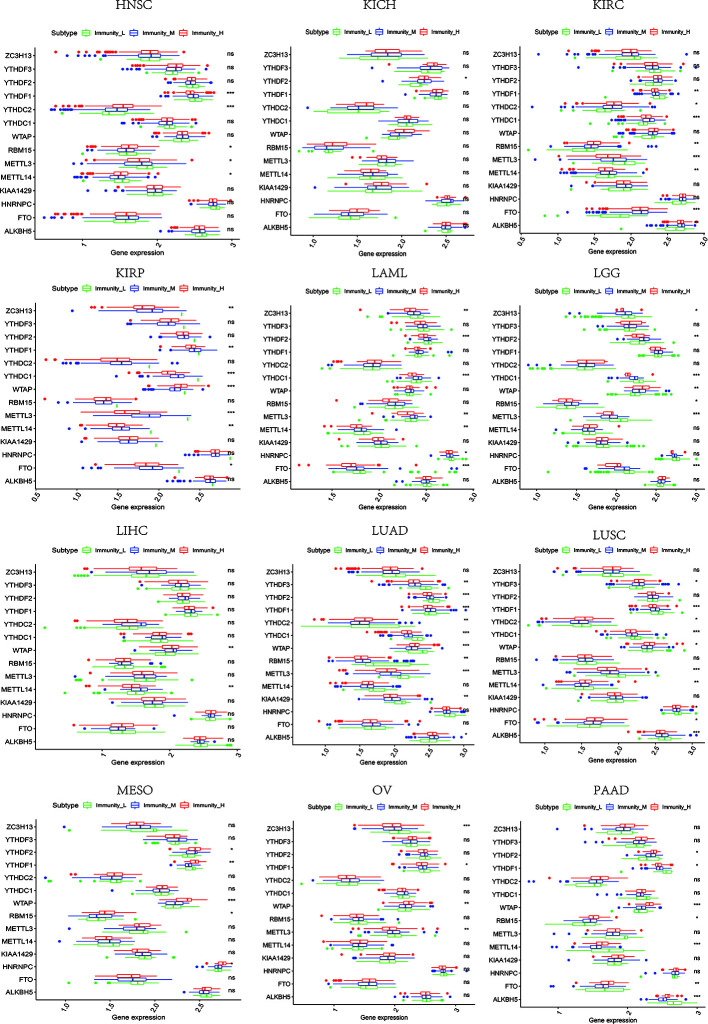
The expression diversity of m^6^A regulators among high, medium, and low immune infiltration levels. * represents P < 0.05, ** represents P < 0.01, *** represents P < 0.001, ns represents P ≥ 0.05.

**Figure 12:**
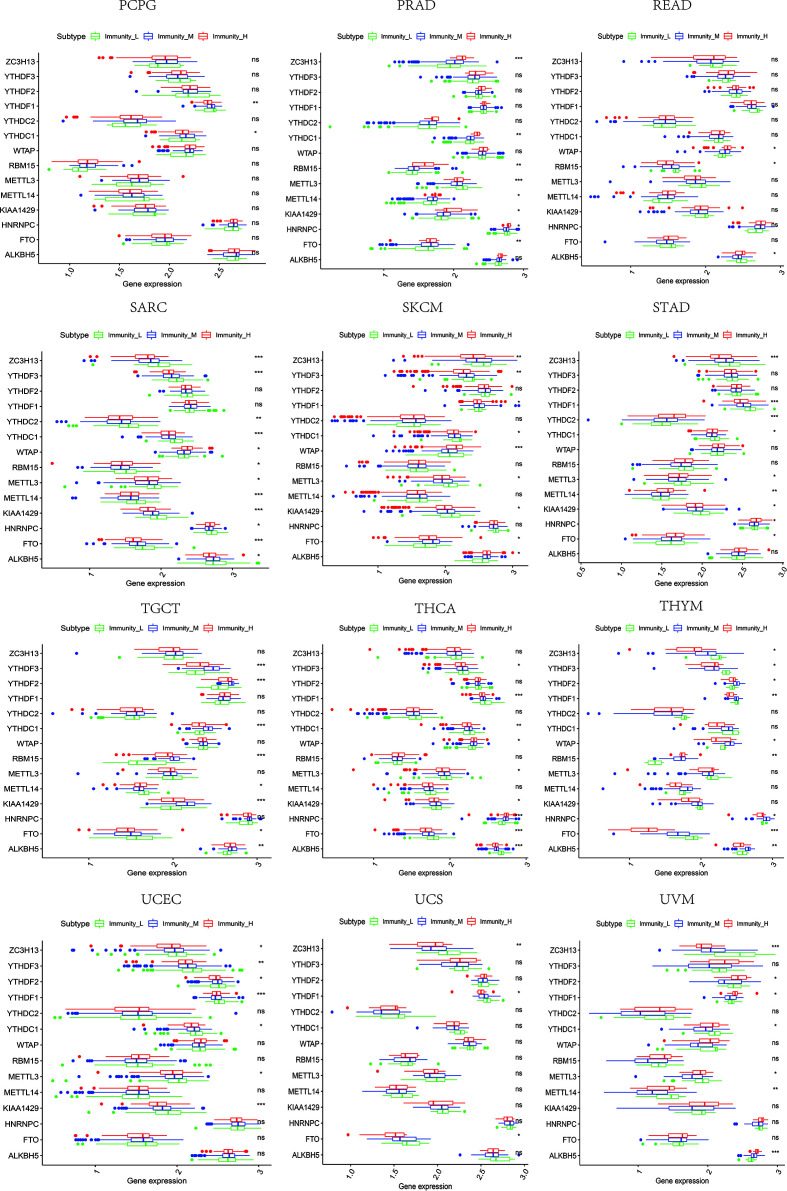
The expression diversity of m^6^A regulators among high, medium, and low immune infiltration levels. * represents P < 0.05, ** represents P < 0.01, *** represents P < 0.001, ns represents P ≥ 0.05.

**Figure 13:**
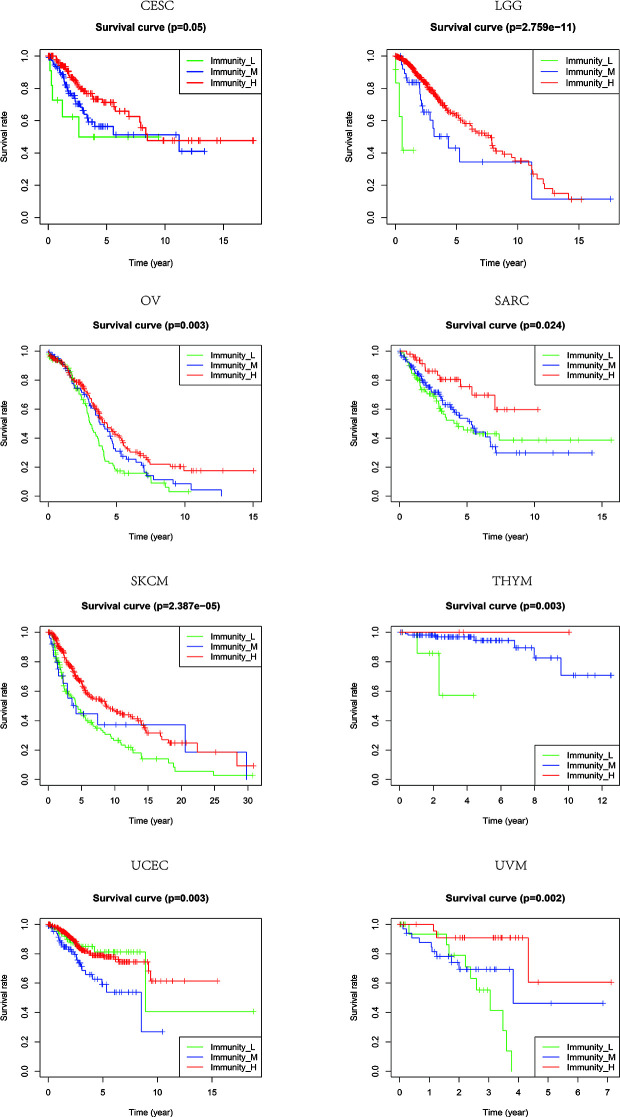
The patients that grouped into high immune infiltration showed better prognosis in CESC, LGG, OV, SARC, SKCM, THYM, UCEC, and UVM.

Based on the ssGSEA scores of infiltrated immune cells, the hierarchical clustering method divided the samples across 33 tumor types into three subgroups as immunity-high, immunity-medium, and immunity-low, representing the density of TILs (**Figures 3**–**5**). Meanwhile, by combining ESTIMATE analysis, we revealed the distribution of immune score, stromal score, ESTIMATE score, and tumor purity between the three immune subgroups. We found that the subgroup’s immune infiltration degree was remarkably consistent with its immune score, stromal score, and ESTIMATE score. Conversely, high immune infiltration, observed in the immunity-high group, was related to low tumor purity, which indicated that we successfully divided all samples into subgroups depending on their TILs and TME characteristics. The next, we found some commonalities between the four IRGs and TILs once again. High immune infiltration was strongly related to high expressions of CD274 (**Figure 6**), CD8A (**Figure 7**), GZMA (**Figure 8**), and PRF1 (**Figure 9**). Almost all the four IRGs showed the rising expression trend with the degree of immune infiltration in 18 tumor types (ACC, BLCA, CESC, CHOL, COAD, DLBC, ESCA, GBM, HNSC, KICH, KIRP, LGG, LIHC, LUAD, LUSC, MESO, OV, PAAD, PRAD, READ, SARC, SKCM, STAD, TGCT, THCA, UCEC, UCS, and UVM). Additionally, we measured the diversity of m^6^A regulators among three subtypes (**Figures 10**–**12**). METTL3 showed vast expression differences in 22 tumor types (ACC, BLCA, BRCA, CESC, CHOL, COAD, GBM, HNSC, KIRC, KIRP, LAML, LGG, LUAD, LUSC, OV, PRAD, SARC, SKCM, STAD, THCA, UCEC, and UVM), although no uniform expression trend was observed among the three subgroups. YTHDF1 and YTHDC1 followed it, showing subgroup-to-group expression varieties in 21 and 19 tumor types. Similarly, no significant expression tendency was observed among subgroups. Besides, high immune infiltration indicated better prognosis in CESC, LGG, OV, SARC, SKCM, THYM, UCEC, and UVM (**Figure 13**). These results showed that m^6^A regulators may affect TILs and result in heterogeneous prognostic outcomes by affecting the expression of IRGs.

### Correlation Between Tumor Mutational Burden and m^6^A Regulators in Pan-Cancer Tissues

**Figure 14:**
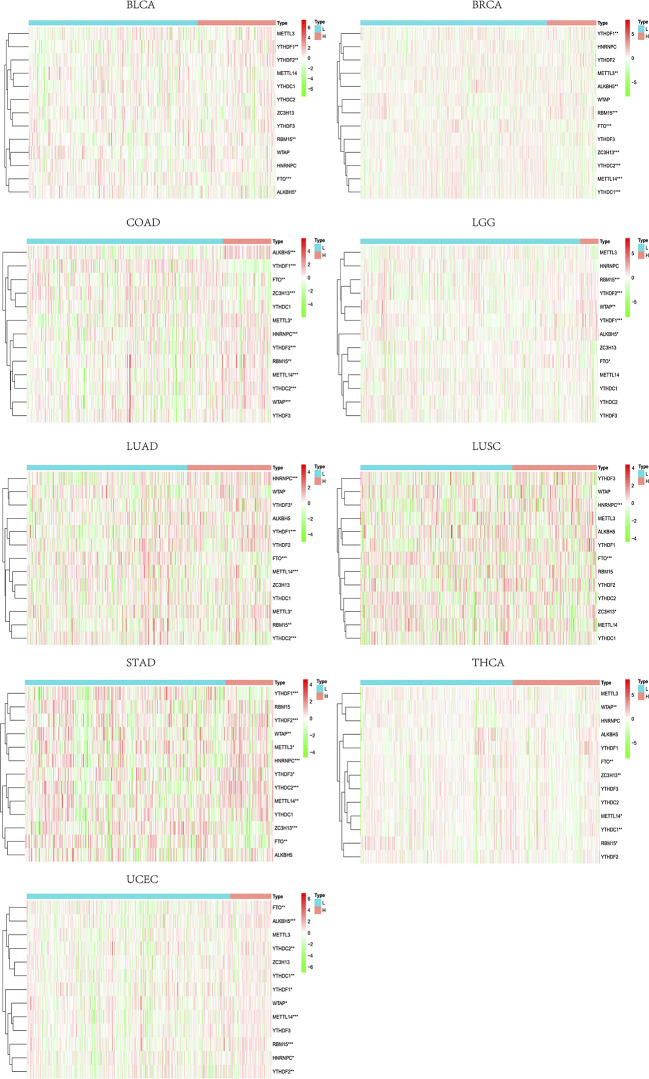
Inter-group differences of m^6^A regulators between high- and low-TMB levels. * represents P < 0.05, ** represents P < 0.01, *** represents P < 0.001.

TMB has been reported to closely influence immunotherapy’s effectiveness across tumor types (8, 37, 38). Tumors with highly mutated burdens are more susceptible to immune cells because of the neoantigens making them respond to ICIs better (8, 39). Considering the close ties between TMB and immune infiltration, TMB could be a predictor of multiple tumors with either anti-CTLA-4 or anti-PD-L1 treatment. Besides, CYT has also been reported to positively correlate with somatic mutations of IRGs (13). Furthermore, high-level co-expression of CD274 and CD8A is usually associated with higher tumor mutation and oncogenic viral infection (14, 40). Nevertheless, we do not know much about the impact of TMB on m^6^A regulators. Since we have demonstrated in the foregoing process that m6A regulators’ impacts on IRGs may extend to the whole TILs and TME, we analyzed the correlation between TMB and m6A regulators in pan-cancer tissues. By taking the median value of TMB, patients’ profiles were divided into high and low TMB groups in each tumor type. It could be seen that large inter-group differences of m^6^A regulators were observed in BLCA, BRCA, COAD, LGG, LUAD, LUSC, STAD, THCA, and UCEC (**Figure 14**). Especially, FTO (in all of nine tumor types), RBM15 (in seven of nine tumor types), and YTHDF1 (in six of nine tumor types) showed a wide range of inter-group expression differences.

### Relationship Between Immune-Related Genes and Clinical Parameters

**Figure 15:**
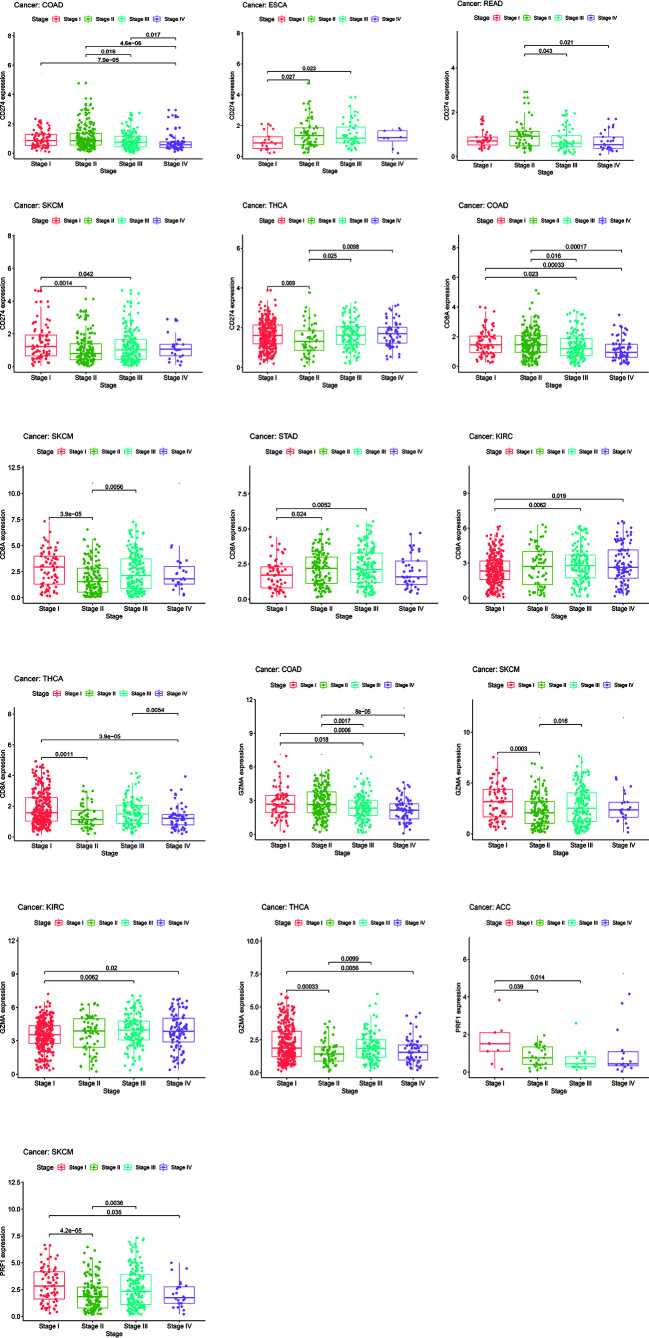
The levels of CD274, CD8A, GZMA, and PRF1 varied between early (stage I and II) and late-stage (stage III and IV) patients.

Chi-square test and Wilcoxon test analyses were performed to explore the associations between clinical parameters and expression levels’ values. The results showed that the level of CD274 varies at tumor stages in COAD, ESCA, READ, SKCM, and THCA, mostly between early (stage I and II) and late-stage (stage III and IV) patients (**Figure 15**). Meanwhile, CD8A and GZMA also had similar expression variants in several tumor types such as COAD, SKCM, STAD, KIRC, and THCA (**Figure 15**). As for PRF1, we only observed this expression differences between stages in ACC and SKCM (**Figure 15**). Overall, the differences of IRGs’ expression levels between early and late-stage patients are more evident in COAD, SKCM, KIRC, THCA, and SKCM. We still used the mean expression value of IRGs as the threshold to compare whether there are differences in OS, DSS, and PFI in various tumor types. The high-expression group of CD274 performed better at OS, DSS, and PFI in ACC and SKCM (**Supplement Figure 15**). The high-expression group of CD8A showed better OS, DSS, and PFI performance in CESC, SKCM, and UCEC (**Supplement Figure 15**). The high-expression group of GZMA had longer survival time, DSS, and PFI in BRCA, SKCM, and UCEC (**Supplement Figure 15**). The high-expression group of PRF1 showed better survival performance or longer PFI in ACC, SKCM, and UCEC (**Supplement Figure 15**).

## Discussion

Immunotherapy has recently received extensive attention and shows efficacy in many cancers (1). However, the variable clinical response has been associated with patients’ immune genomic characteristics as much as other features such as TME and TILs (7, 9, 41, 42). Due to the regulation of the m^6^A, modifications are associated with almost any step of mRNA metabolism. There is convincing evidence that m6A modification is particularly critical in a variety of pathological and physiological immune responses, including T cell homeostasis and differentiation, dendritic cell activation (43–45).

Our study intends to prove that m^6^A regulators could alter TME properties by influencing key IRGs and TILs. Based on the previous researches, CD274, CD8A, GZMA, and PRF1 were selected for further analysis as being iconic targets of ICIs and key genes affecting TILs. CD274, also known as PD-L1, was found to have high expression on the surface of various tumor cells (46, 47), involving the development of tumors and affecting response to ICIs’ treatment and clinical outcomes (48). CD8A encodes part of cell surface glycoprotein on most cytotoxic T lymphocyte, which includes adaptive immune response-induced CD8+ cytolytic T cells (49, 50), plays a crucial role in the antitumor activity of anti-PD-L1 (51). GZMA and PRF1 serve as two key cytolytic effectors, which are proved to bond with CD8+ T cell activation and affect clinical responses to ICIs (13). All four IRGs were proved to have impacts on pathological stages and clinical outcomes. Furthermore, they showed similar TILs characteristics, of which it is worth noting that the expression tendency of T cells CD8, T cells CD4 memory activated is consistent with the expression levels of all four immune-related genes. Not surprisingly, ICIs had been proved to rely heavily on functional T cells CD8 (CD8+ T cells) (52). Moreover, activated memory CD4 T cells also play a crucial role in effective antitumor immunity (53). And ssGSEA analyses revealed expression levels of CD274, CD8A, GZMA, and PRF1 were positively correlated with high immune infiltration in 18 tumor types, which was consistent with the result of ESTIMATE analyses that all the four IRGs were positively expressed with the immune score or stromal score in almost all of 33 tumor types. High immune infiltration and high-level of the four IRGs were found to predict better prognosis in multiple tumor types like CESC, SKCM, THYM, and UCEC. So far, we believed that the four IRGs could represent the TILs characteristics of tumor tissues and to be used to classify TME. Based on the co-expression correlation between CD274 and CD8A in 29 tumor types, and between GZMA and PRF1 in 32 tumors, we further classified TME into eight groups according to IRGs’ expression level. The exact opposite expression tendency of m6A regulators was found among the subgroups of type I to type VIII, suggesting that m6A regulators may be essential for phenotypic modifications of IRGs. To further confirm this correlation, the ssGSEA method was used to classify immuno-subtypes and genomic expression diversities. IRGs and m^6^A regulators were proved to vary with immuno-subtypes in different TME characteristics.

Neoantigens are carried by highly mutated tumors, which are susceptible to immune cells and own a better response to ICIs (38). Previous studies have shown that TMB could predict patients’ survival in diverse tumor types with either anti-CTLA-4 or anti-PD-1 treatment (8, 39). We further evaluated the correlation between TMB and m^6^A regulators. In nine tumor types (BLCA, BRCA, COAD, LGG, LUAD, LUSC, STAD, THCA, and UCEC), m^6^A regulators’ expressions also differ along with TMB levels. In conclusion, we have demonstrated the prevalent genetic expression alterations of the crucial IRGs are related to m^6^A regulators across tumor types. Both of IRGs and m^6^A regulators are tightly correlated with TME characteristics and TILs features. Our systematic and comprehensive analyses in the landscape of molecular alterations and clinical relevance provide a foundation for understanding the internal mechanisms of TME and its overall prognosis and the development of potential therapeutic targets.

## Data Availability Statement

Publicly available datasets were analyzed in this study. These data can be found here: The original contributions presented in the study are publicly available in TCGA database (https://portal.gdc.cancer.gov/). This data can be found in the UCSC Xena browser (https://xenabrowser.net).

## Author Contributions

JZ, JX, MW, and DH handled the conceptualization and methodology. JZ, MW, and JX handled the software. JX and DH handled the validation. JZ and MW were in charge of the original draft preparation. JX and DH were responsible for the review and editing. The first authors of this manuscript are JZ and JX. These authors contributed equally to this work. All authors contributed to the article and approved the submitted version.

## Funding

This work was supported by the Science and Technology Project in Tongliang District (No.TL2019-25).

## Conflict of Interest

The authors declare that the research was conducted in the absence of any commercial or financial relationships that could be construed as a potential conflict of interest.

## Supplementary Material

Supplementary Figure 1 Pancancer TILs component analyses of CD274 *via* CIBERSORT.

Supplementary Figure 2 Pancancer TILs component analyses of CD8A *via* CIBERSORT.

Supplementary Figure 3 Pancancer TILs component analyses of GZMA *via* CIBERSORT.

Supplementary Figure 4 Pancancer TILs component analyses of PRF1 *via* CIBERSORT.

Supplementary Figure 5 Correlation analysis between CD274 and ESTIMATE immune score or stromal score across pancancer tissues.

Supplementary Figure 6 Correlation analysis between CD8A and ESTIMATE immune score or stromal score across pancancer tissues.

Supplementary Figure 7 Correlation analysis between GZMA and ESTIMATE immune score or stromal score across pancancer tissues.

Supplementary Figure 8 Correlation analysis between PRF1 and ESTIMATE immune score or stromal score across pancancer tissues.

Supplementary Figure 9 Expression analysis of m^6^A regulators between high- and low-expression levels of CD274.

Supplementary Figure 10 Expression analysis of m^6^A regulators between high- and low-expression levels of CD8A.

Supplementary Figure 11 Expression analysis of m^6^A regulators between high- and low-expression levels of GZMA.

Supplementary Figure 12 Expression analysis of m^6^A regulators between high- and low-expression levels of PRF1.

Supplementary Figure 13 Co-expression relationship between CD274 and CD8A across pancancer tissues.

Supplementary Figure 14 Co-expression relationship between GZMA and PRF1 across pancancer tissues.

Supplementary Figure 15 The high-expression level of CD274 performed better at OS, DSS, and PFI in ACC and SKCM; The high-expression level of CD8A showed better OS, DSS, and PFI performance in CESC, SKCM, and UCEC; The high-expression level of GZMA had longer survival time, DSS, and PFI in BRCA, SKCM, and UCEC; The high-expression level of PRF1 showed better survival performance or longer PFI in ACC, SKCM, and UCEC.
