# Supplementary figures and images for: Pan-Cancer Molecular Characterization of m6A Regulators and Immunogenomic Perspective on the Tumor Microenvironment
Source: Front Oncol. 2021 Jan 28;10:618374. doi: 10.3389/fonc.2020.618374 (PMC7876474; doi:10.3389/fonc.2020.618374)

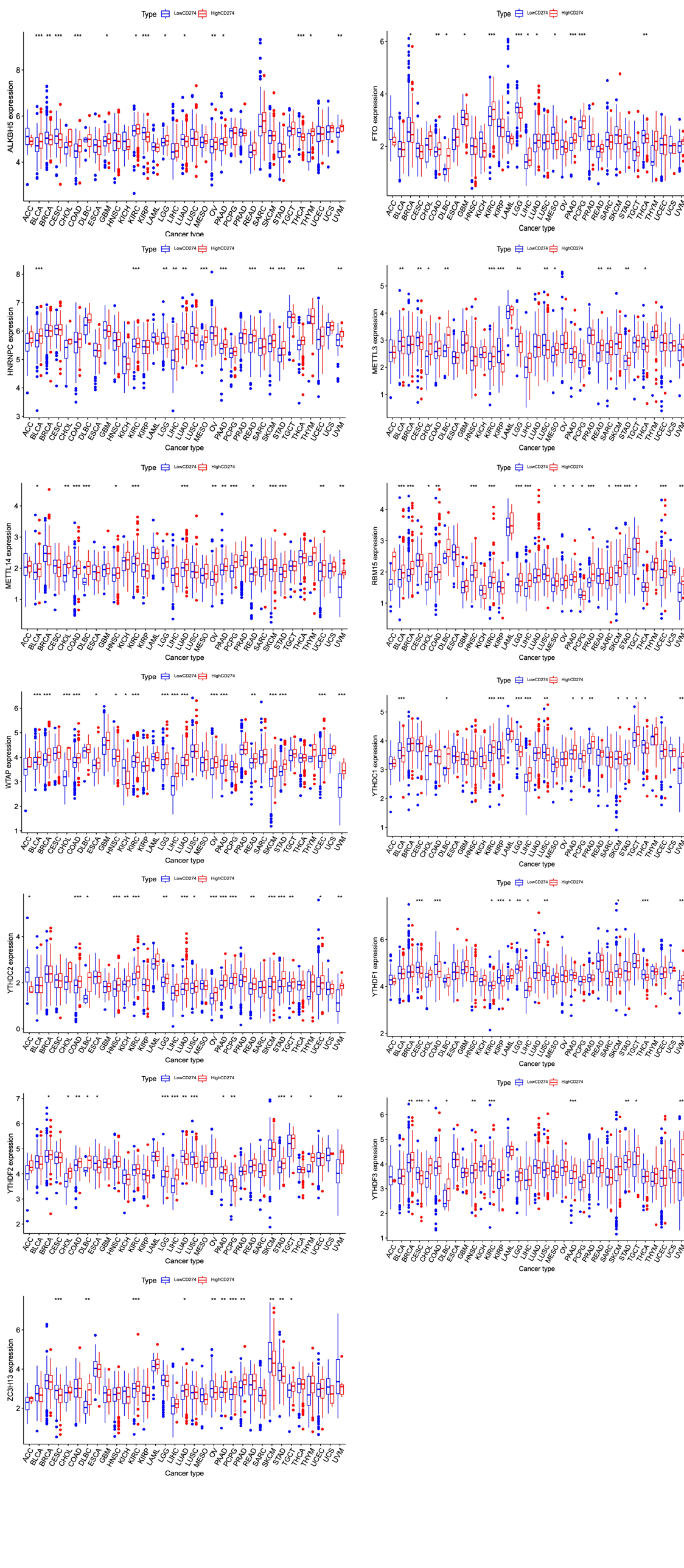

Supplement: Supplementary Figure 9 — Expression analysis of m6A regulators between high- and low-expression levels of CD274. [file Image_9.jpeg]

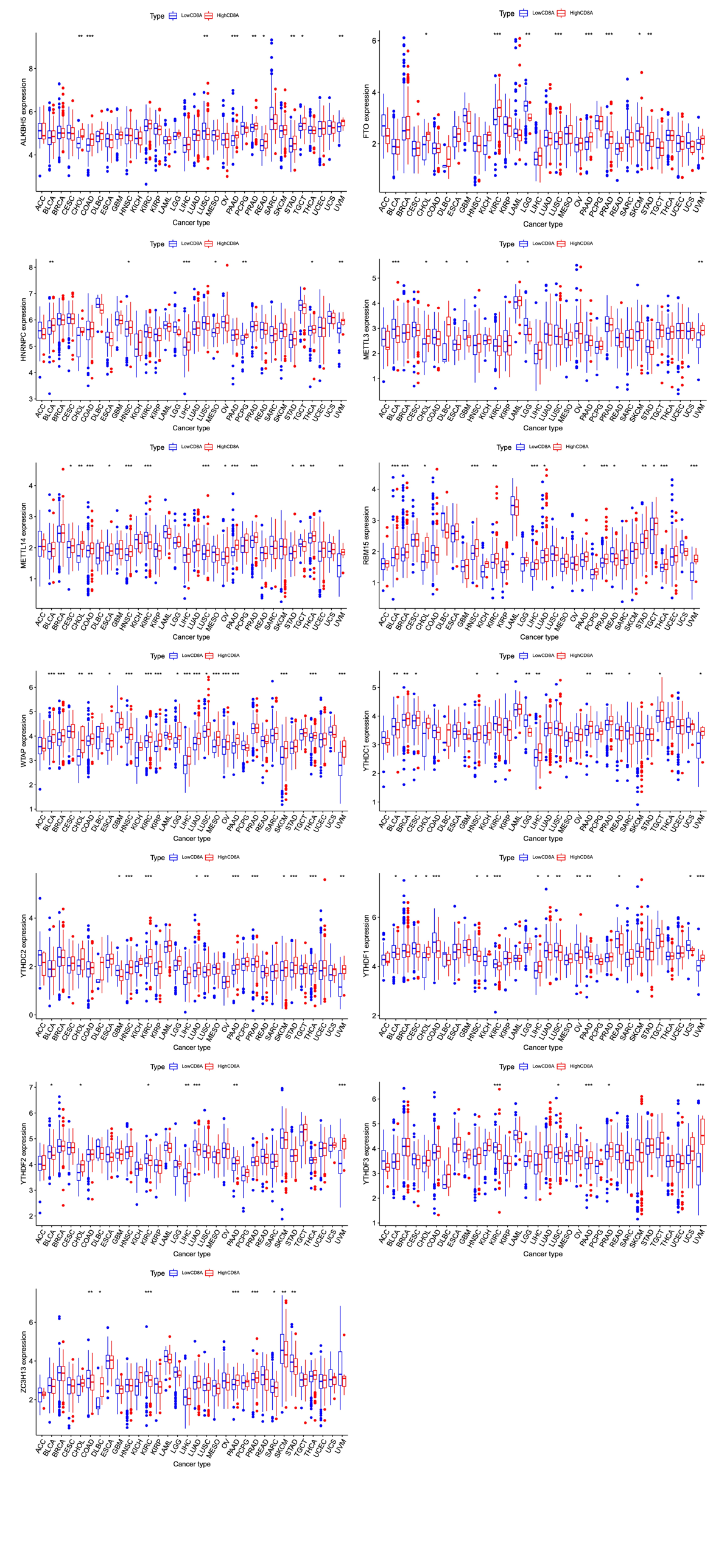

Supplement: Supplementary Figure 10 — Expression analysis of m6A regulators between high- and low-expression levels of CD8A. [file Image_10.jpeg]

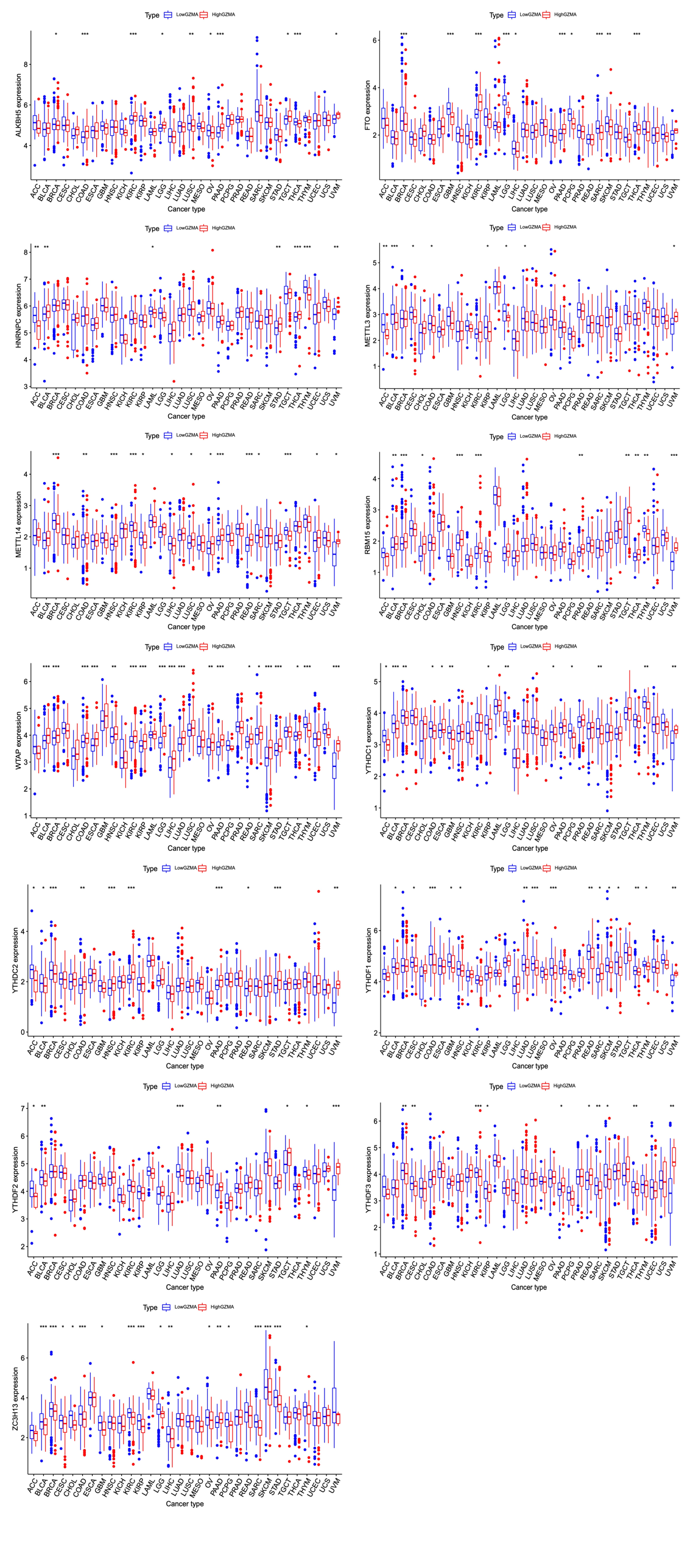

Supplement: Supplementary Figure 11 — Expression analysis of m6A regulators between high- and low-expression levels of GZMA. [file Image_11.jpeg]

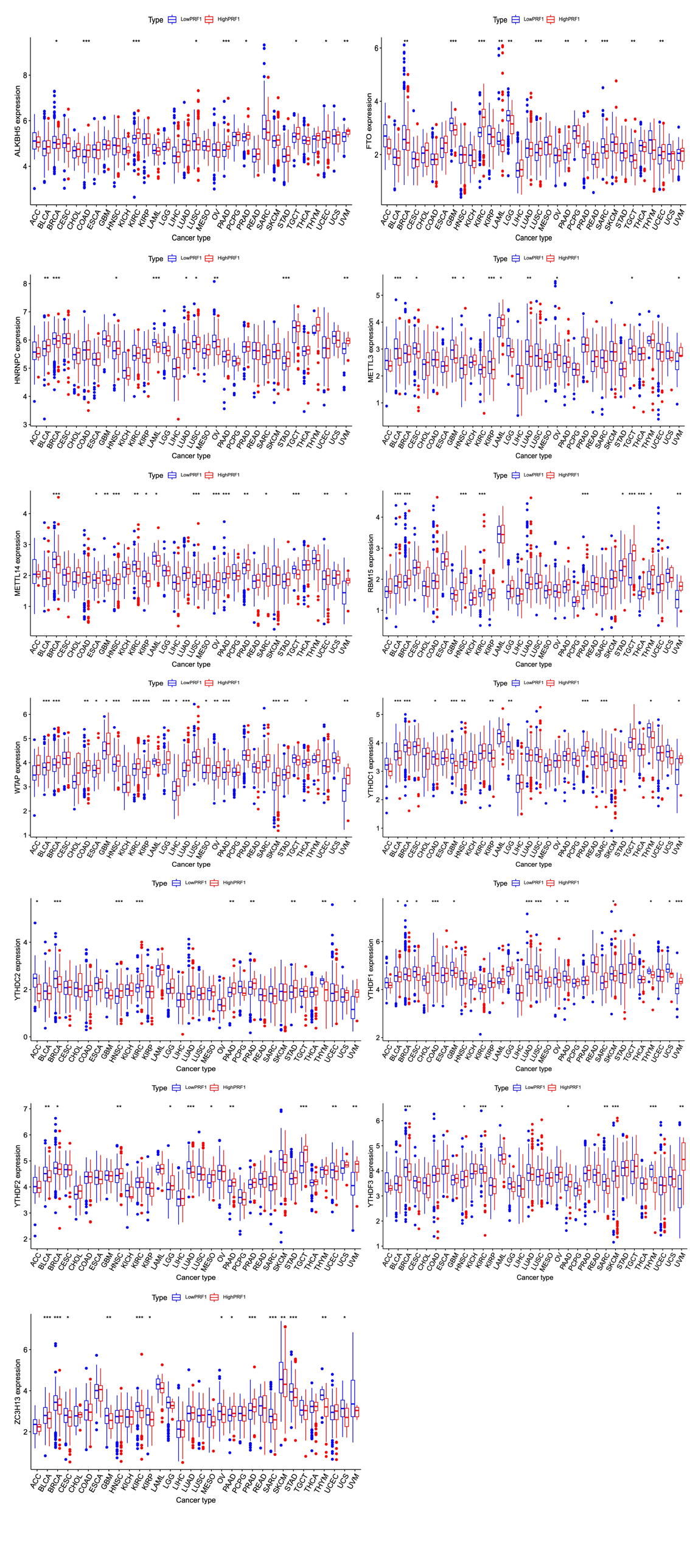

Supplement: Supplementary Figure 12 — Expression analysis of m6A regulators between high- and low-expression levels of PRF1. [file Image_12.jpeg]

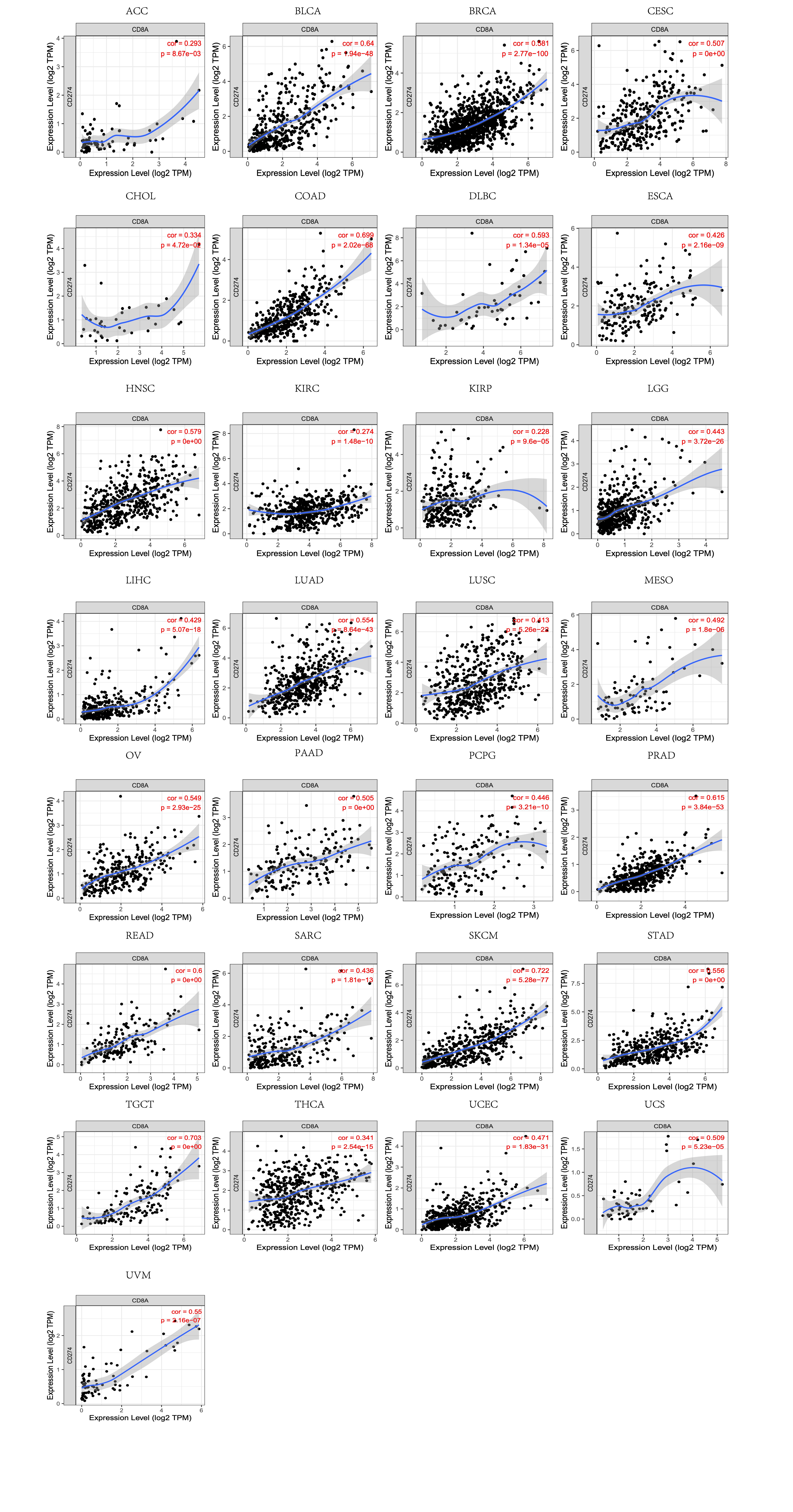

Supplement: Supplementary Figure 13 — Co-expression relationship between CD274 and CD8A across pancancer tissues. [file Image_13.jpeg]

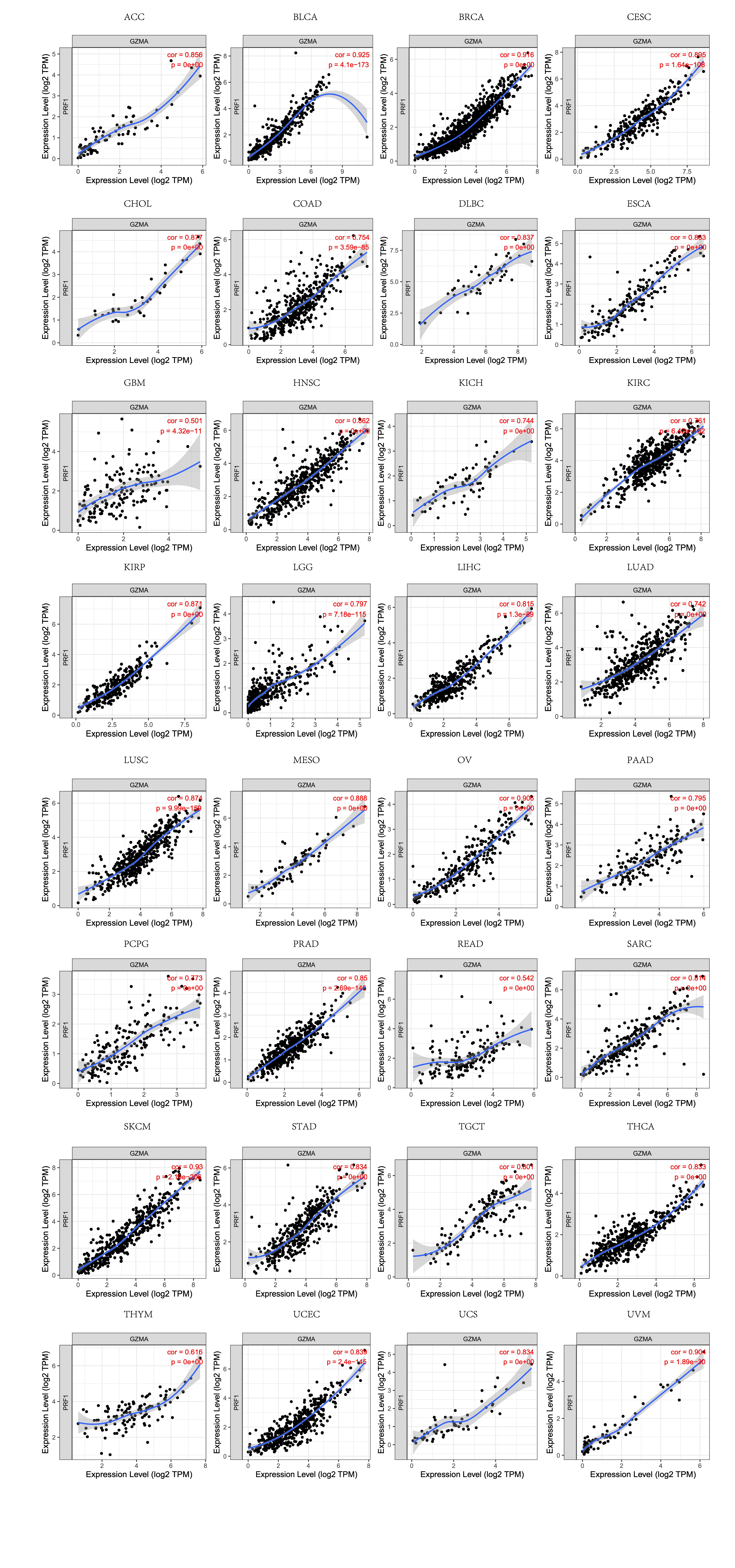

Supplement: Supplementary Figure 14 — Co-expression relationship between GZMA and PRF1 across pancancer tissues. [file Image_14.jpeg]
